# Introduction into Nigeria of a Distinct Genotype of Avian Influenza Virus (H5N1)

**DOI:** 10.3201/eid1503.081161

**Published:** 2009-03

**Authors:** Alice Fusaro, Tony Joannis, Isabella Monne, Annalisa Salviato, Bitrus Yakubu, Clement Meseko, Tinuke Oladokun, Sonia Fassina, Ilaria Capua, Giovanni Cattoli

**Affiliations:** Istituto Zooprofilattico Sperimentale delle Venezie, Padova, Italy (A. Fusaro, I. Monne, A. Salviato, S. Fassina, I. Capua, G. Cattoli); National Veterinary Research Institute, Vom, Nigeria (T. Joannis, B. Yakubu, C. Meseko, T. Oladokun)

**Keywords:** Highly pathogenic avian influenza virus, H5N1, Nigeria, phylogenetic analysis, dispatch

## Abstract

Genetic characterization of highly pathogenic avian influenza viruses (H5N1) isolated in July 2008 in Nigeria indicates that a distinct genotype, never before detected in Africa, reached the continent. Phylogenetic analysis showed that the viruses are genetically closely related to European and Middle Eastern influenza A (H5N1) isolates detected in 2007.

In February 2006, highly pathogenic avian influenza (HPAI) virus of the H5N1 subtype was detected in chickens in Kaduna state in northern Nigeria, the first African country reporting a confirmed HPAI (H5N1) outbreak. The infection later spread to 25 of the 36 Nigerian states and to the Federal Capital Territory and persisted for 21 months. Consequently, ≈368,000 domestic birds (mostly chickens; also guinea fowl, turkeys, ducks, geese, and ostriches) were killed by the virus or culled, and a fatal human case was reported. By the end of 2007, the outbreaks appeared to have been controlled by such measures as stamping-out with compensation, restricting movement of poultry and poultry products, improving biosecurity measures, and enhanced surveillance systems. The last reported case of HPAI occurred in the southern state of Anambra and was reported by the Government of Nigeria in October 2007. In addition to the routine avian influenza surveillance program, industrial poultry in the 36 states plus the Federal Capital Territory, and live bird markets have been actively monitored since March 2007, with >13,000 samples collected and analyzed for avian influenza viruses (T. Joannis, pers. comm.).

During the surveillance activities at the live bird markets, new cases of HPAI (H5N1) were detected in July 2008 in the city of Gombe in the northeastern state of Gombe after a 9-month period during which no influenza A virus was identified. In particular, 2 tracheal swabs collected from apparently healthy domestic ducks were submitted to the laboratory for virus isolation in embryonated specific antibody-negative fowl eggs. Allantoic fluid harvested from inoculated eggs showing embryo death tested positive for hemagglutinating agents. RNA extracted from positive allantoic fluid also tested positive by real-time reverse transcription–PCR for type A influenza RNA (*1*) and for the H5 subtype (*2*). Hemagglutination and neuraminidase (NA) inhibition assays with monospecific antiserum (*3*) confirmed the H5N1 subtype. Viruses were designated as A/duck/Nigeria/3724-2/2008 and A/duck/Nigeria/3724-10/2008.

## The Study

We obtained the full-length genome sequence for A/duck/Nigeria/3724-2/2008 and the sequence of the hemagglutinin (HA) segment for A/duck/Nigeria/3724-10/2008 (*4*). Sequences of the 8 gene segments of A/duck/Nigeria/3724-2/2008 were submitted to the Global Initiative on Sharing Avian Influenza Data public database (accession nos. EPI161701–EPI161708). The HA segment of the 2 isolates was identical, and the deduced amino acid sequence of the HA cleavage site was characteristic of HPAI (H5N1) (PQGERRRKKR*GLF). The highly pathogenic pathotype was confirmed by the result of the intravenous virus pathogenicity index test (index 2.87) (*3*).

**Figure 1:**
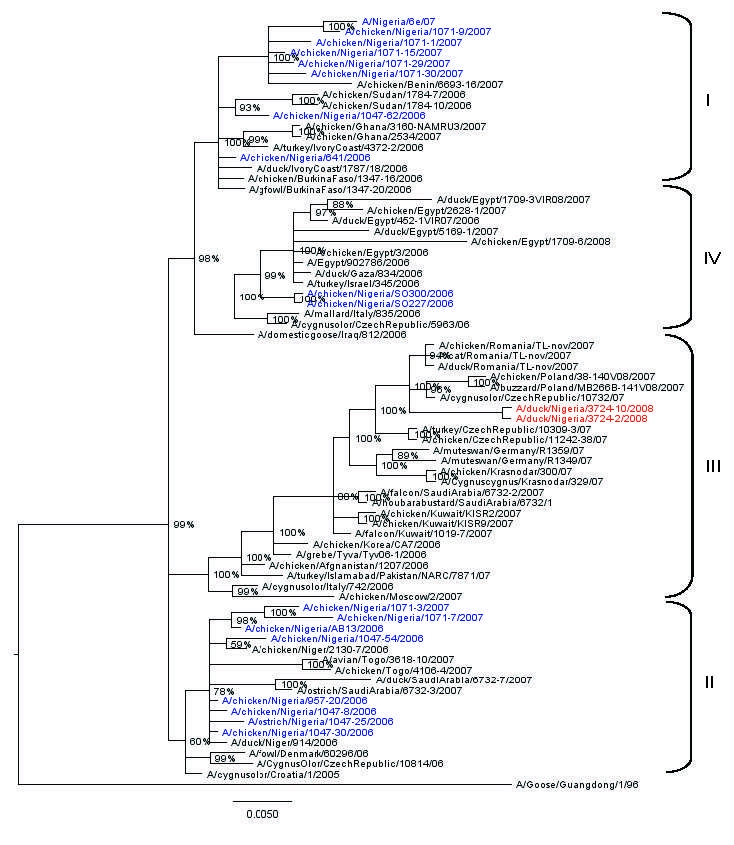
Phylogenetic tree constructed by Bayesian analysis of the hemagglutinin gene segment of representative influenza viruses A (H5N1) from Africa, Europe, and the Middle East. Taxon names of the Nigerian viruses isolated during 2006–2007 are marked in blue, 2008 isolates in red. Posterior probabilities of the clades are indicated above the nodes. Scale bar indicates number of nucleotide substitutions per site.

**Figure 2:**
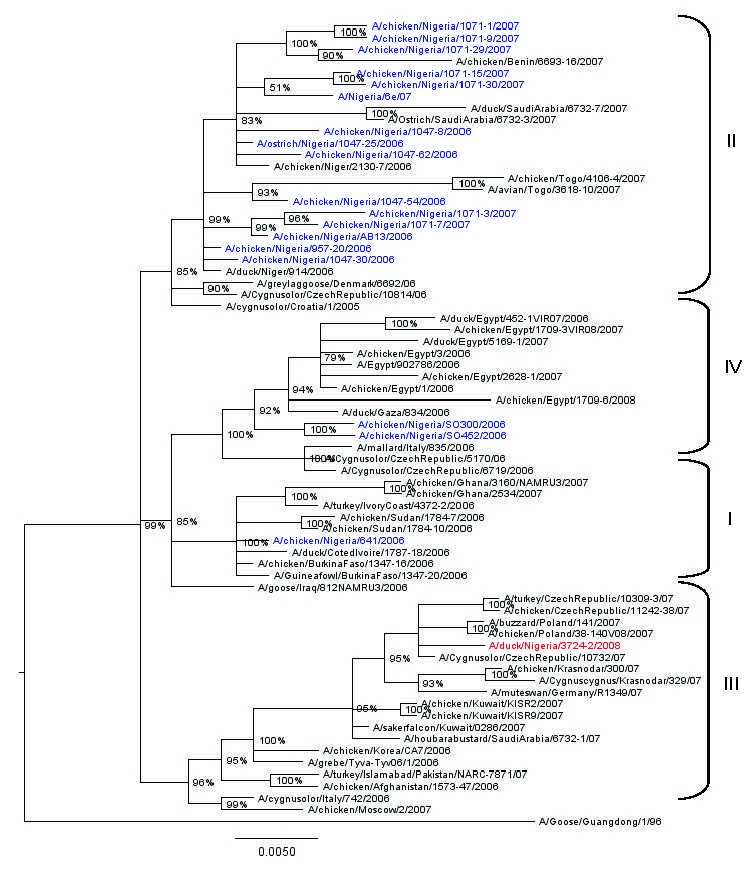
Phylogenetic tree constructed by Bayesian analysis of the neuraminidase gene segment of representative influenza viruses A (H5N1) from Africa, Europe, and the Middle East. Taxon names of the Nigerian viruses isolated during 2006–2007 are marked in blue, 2008 isolate in red. Posterior probabilities of the clades are indicated above the nodes. Scale bar indicates number of nucleotide substitutions per site.

Phylogenetic analysis of the 8 genes was conducted by using MEGA 4 (*5*) with the neighbor-joining method, and the HA and NA tree topology was confirmed by using Bayesian methods (*6*) (Figures 1, 2). Phylogenetic analysis of the HA gene segment showed the viruses fall in clade 2.2, according to the unified nomenclature system (*7*). Unexpectedly, the viruses were grouped separately from the viruses previously detected in Nigeria and in other African countries. They clustered in the sublineage here designated III, together with HPAI (H5N1) viruses isolated in 2007 in Europe and Middle East (Figure 1). Phylogenetic analysis of the NA gene segment of A/duck/Nigeria/3724-2/2008 supported these results (Figure 2).

Sequence analysis of the 8 gene segments of A/duck/Nigeria/3724-2/2008 showed the highest similarity at the nucleotide level with the HPAI (H5N1) virus isolate A/*Cygnus olor*/Czech Republic/10732/2007 (99.3% for HA, 99.8% for NA, 99.7% for nonstructural protein, 99.4% for polymerase basic protein 1 [PB1], 99.7% for polymerase basic protein 2 [PB2], 99.8% for nucleoprotein [NP], 99.3% for polymerase acidic protein [PA], and 100% for matrix [MA] protein) and with the HPAI (H5N1) strains from Romania collected in 2007 (99.3%, only HA gene segment is available). For the HA protein, only 3 amino acids differences were observed between the Nigerian isolate and the strain A/*Cygnus olor*/Czech Republic/10732/2007. Lower similarities (ranging from 96.9% to 98% for the HA gene) were shown with previous isolates from Nigeria. No molecular changes were associated with increased affinity toward α 2,6 linkage sialic acid substrates in the HA receptor-binding domain (*8*) or mutations related to resistance to NA inhibitors and to adamantanes were observed in the HA, NA, and M2 genes of the Nigerian isolate. Analysis of the amino acid sequences of the internal proteins of A/duck/Nigeria/3724-2/2008 virus showed the amino acid lysine at position 627 of the PB2 gene known to be associated with increased virulence of HPAI (H5N1) virus in mice (*9*) and 1 amino acid mutation at position 33 (V33I) of the NP gene, which is described as genetic signature of human influenza A virus (*10*). The PB1-F2 protein had 1 mutation at position 66 (N66S), previously observed only in the Hong Kong 1997 subtype H5N1 viruses and in the 1918 pandemic strain (A/Brevig Mission/18) and is associated to high pathogenic phenotype in mice (*11*).

## Conclusions

Since the earliest known progenitor detected in China, A/goose/Guandong/96, numerous lineages of HPAI (H5N1) viruses have emerged (*7*). Introduction and spread of distinct H5N1 genetic lineages were described in several Asian countries and in Europe, such as Germany, Italy, and France (*12*,*13*). Surprisingly, Nigeria is the only country in Africa where HPAI (H5N1) belonging to distinct sublineages have been detected (*4*,*12*,*14*). Previous genetic characterization of HPAI (H5N1) viruses isolated during 2006 and 2007 indicated the cocirculation in Nigeria of 3 distinct sublineages, here designated I, II, and IV (*4*,*14*). Sublineages I and II appeared to be widespread in this country during 2006 and 2007 (*14*), and their extended cocirculation enabled reassortment events between these sublineages. The first reassortant virus was identified in June 2006 (*12*), and in early 2007, an additional reassortant virus was identified in 7 of 22 Nigerian states where infection was found (*4*).

Our results indicate a novel introduction in Nigeria of a virus belonging to sublineage III, a genotype not previously detected in the African continent. Indeed, previous surveillance efforts (passive and active) since 2006 in Nigeria and other African countries (*4*,*12*,*14*) never showed evidence of the circulation of a virus belonging to this sublineage. Viruses belonging to sublineage III have been detected in domestic and wild birds in 2007 in European, Middle Eastern, and Asian countries such as Germany, Poland, Romania, the Czech Republic, Kuwait, Saudi Arabia, Russia, and Pakistan.

The Nigerian isolate A/duck/Nigeria/3724-2/2008 resulted in a genome almost identical to an isolate from a mute swan living in the wild, namely, A/*Cygnus olor*/Czech Republic/10732/2007 (*15*). This finding, however, does not shed light on how the virus was introduced into Nigeria because neither of the 2 main means of spread, through wild birds or the poultry trade, can be excluded.

The evidence of a mutation in the PB1-F2 gene segment that increases the pathogenicity of the virus in mammalian hosts is of concern. This type of mutation has never been observed in HPAI (H5N1) viruses of clade 2.2; this finding supports the need for a continuous monitoring effort of influenza viruses A (H5N1) viral genotypes and their evolution. Our findings highlight the evolving epidemiology of HPAI (H5N1) viruses and the need for implementation and maintenance of sustainable surveillance programs in countries where infection has been found and in countries where it has not. The outcome of these efforts, however, can be maximized only through prompt reporting to international organizations and international collaboration that leads to timely molecular and antigenic comparisons of isolates.
